# Alogliptin/Amentoflavone Combination Mitigates Bleomycin-Induced Lung Fibrosis: The Role of Oxidative Stress, TXNIP-Mediated Pyroptosis, and Autophagy/Apoptosis Balance

**DOI:** 10.3390/ph19060822

**Published:** 2026-05-24

**Authors:** Hanan Abdelmawgoud Atia, Hemat A. Elariny, Gehad M. Subaiea, Asmaa Saleh, Amany M. Khalifa, Doaa Hellal, Kareem M. Younes, Ahmed M. Kabel

**Affiliations:** 1 Department of Pharmacology and Toxicology, College of Pharmacy, University of Ha’il, Ha’il 55473, Saudi Arabia; 2 Department of Pharmaceutical Sciences, College of Pharmacy, Princess Nourah bint Abdulrahman University, Riyadh 11671, Saudi Arabia; 3 Department of Pathology, College of Medicine, University of Ha’il, Ha’il 55473, Saudi Arabia; 4 Department of Pharmacology, College of Medicine, University of Ha’il, Ha’il 55473, Saudi Arabia; 5 Department of Pharmaceutical Chemistry, College of Pharmacy, University of Ha’il, Ha’il 55473, Saudi Arabia; 6 Pharmacology Department, Faculty of Medicine, Tanta University, Tanta 31527, Egypt

**Keywords:** alogliptin, amentoflavone, bleomycin, lung fibrosis, pyroptosis, mice

## Abstract

**Background/Objectives:** Bleomycin is an antineoplastic antibiotic used in the management of various malignancies. Nevertheless, its benefits are constrained by the development of pulmonary fibrosis. Amentoflavone, a biflavonoid, exhibits diverse pharmacological activities, including anti-inflammatory, antiviral, antioxidant, and antitumor effects, whereas alogliptin possesses antioxidant and anti-inflammatory properties. This study aimed to assess the potential protective effects of alogliptin and/or amentoflavone in a murine model of bleomycin-induced pulmonary fibrosis and to clarify the underlying mechanisms. **Methods:** Fifty male C57BL/6 mice were randomly divided into 5 equal groups: control, bleomycin, bleomycin + alogliptin, bleomycin + amentoflavone, and bleomycin + alogliptin + amentoflavone. The assessed endpoints included lung weight/body weight index, lung tissue fibrotic mediators, oxidative stress parameters, proinflammatory cytokines, and pyroptotic and autophagy mediators. Also, the bronchoalveolar lavage fluid (BALF) was evaluated for total and differential leukocytic counts and lactate dehydrogenase (LDH) activity. Moreover, vascular responses to potassium chloride, phenylephrine, and carbachol, together with tracheal responses to carbachol were determined. Lung tissues were further examined histopathologically and immunohistochemically. **Results:** Treatment with alogliptin and/or amentoflavone significantly decreased the lung weight/body weight index and BALF LDH activity, concomitant with mitigation of lung tissue oxidative stress parameters, fibrotic mediators, apoptosis, and pyroptosis with a significant augmentation of autophagy signals, alongside marked improvement in the lung architecture and vascular and airway reactivity compared with the bleomycin group. These effects were most pronounced with animals treated with the alogliptin/amentoflavone combination. **Conclusions:** These findings suggest that combined alogliptin and amentoflavone may constitute a promising strategy to prevent bleomycin-induced lung fibrosis.

## 1. Introduction

Pulmonary fibrosis is a complex respiratory disorder characterized by progressive swelling and scarring of the alveoli and interstitial tissues of the lung, leading to impaired gas exchange and respiratory failure [1]. Diverse triggers have been reported to develop lung fibrosis, including viral infections, thoracic radiotherapy, chemotherapeutic agents such as bleomycin, environmental toxins, and connective tissue diseases like rheumatoid arthritis and systemic sclerosis [2]. Central to disease initiation is recurrent alveolar epithelial cell injury followed by dysregulated wound repair, with persistent fibroblast/myofibroblast activation, excessive extracellular matrix (ECM) deposition, and inadequate matrix degradation [3]. Myofibroblasts amplify profibrotic cytokine signaling, particularly transforming growth factor beta 1 (TGF β1), driving collagen accumulation and the upregulation of tissue inhibitor metalloproteinases, which collectively prevent further degradation of collagen and increased deposition of ECM proteins [3].

Bleomycin (BLM), a chemotherapeutic glycopeptide antibiotic, is commonly included in regimens for lymphomas as well as certain solid cancers such as testicular and breast carcinoma [4]. Clinically and experimentally, BLM administration is associated with marked alterations in lung structure and biochemistry that predispose to pulmonary fibrosis [5]. This toxicity is linked to BLM-induced reactive oxygen species (ROS) formation, which binds to and cleaves DNA, leading to DNA damage that initiates robust inflammatory and fibrogenic responses [6]. Concurrently, by depleting the key components of the cellular antioxidant machinery, BLM exacerbates oxidative injury and accelerates fibrotic remodeling of the lung parenchyma [7].

Thioredoxin-interacting protein (TXNIP) is a cellular regulator that binds to and inhibits thioredoxin, a key antioxidant protein [8]. Consequently, TXNIP interferes with the cellular ability to neutralize ROS, thereby promoting oxidative stress [9]. Furthermore, TXNIP plays a key role in metabolic dysfunction, inflammation, and pathways that regulate cellular death [10]. In bleomycin-induced pulmonary fibrosis, TXNIP acts as a critical link between oxidative stress and inflammatory cell death [11]. When bleomycin damages the pulmonary tissues, TXNIP expression rises and disrupts the antioxidant defenses provided by thioredoxin, creating a pro-oxidant environment [12]. Under these conditions, TXNIP binds to and activates the NOD-like receptor protein 3 (NLRP3) inflammasome, which triggers caspase-1 activation and the cleavage of gasdermin D, leading to pyroptosis [13]. This form of programmed inflammatory cell death releases proinflammatory cytokines such as IL-1β and IL-18, amplifying local inflammation and driving fibroblast activation [14,15]. The combined effects of oxidative stress, pyroptotic cell injury, and persistent inflammation promote excessive collagen deposition, making TXNIP-mediated pyroptosis a central mechanism in the progression of bleomycin-induced pulmonary fibrosis [16].

Alogliptin is a member of the dipeptidyl peptidase 4 (DPP4) inhibitors, a class of oral antidiabetic drugs, and has been approved by the U.S. Food and Drug Administration for the management of type 2 diabetes mellitus [17]. By selectively inhibiting DPP 4, alogliptin slows the enzymatic degradation of endogenous incretin hormones, mainly glucagon-like peptide 1 (GLP 1) and glucose-dependent insulinotropic polypeptide (GIP), which in turn enhances glucose-dependent insulin secretion from pancreatic β cells while suppressing glucagon release and hepatic gluconeogenesis, thereby improving overall glycemic control without substantially increasing the risk of hypoglycemia [18]. Beyond its glucose-lowering action, alogliptin has a variety of antioxidant and anti-inflammatory effects that seem to contribute to its cytoprotective effects [19]. Experimental and clinical evidence regarding DPP-4 inhibitors demonstrates decreases in oxidative stress and pro-inflammatory cytokines, alongside enhancements in endothelial and tissue functions across various organs [20,21]. There is strong evidence that DPP-4 inhibition has a strong antifibrotic effect because DPP-4 interacts bidirectionally with transforming growth factor-β1 (TGF-β), which is a key profibrotic cytokine that activates fibroblasts [22]. Through modulation of the TGF β/DPP 4 axis, alogliptin may inhibit endothelial to mesenchymal transition (EndMT), a process in which endothelial cells acquire mesenchymal/myofibroblast features and contribute to the pool of activated fibroblasts within damaged tissues [23].

Amentoflavone is a naturally occurring biflavonoid identified in numerous plant species, including *Ginkgo biloba*, *Hypericum perforatum*, *Selaginella tamariscina*, *Chamaecyparis obtuse*, *Biophytum sensitivum*, and *Xerophyta plicata* [24]. Amentoflavone has recently garnered increasing attention as a bioactive compound exhibiting a broad range of pharmacological effects that could benefit various organ systems [25]. These effects include modulation of the key mediators of inflammation, enhancement of endogenous antioxidant defense pathways, and attenuation of fibrotic remodeling across diverse body tissues [26,27,28]. Recent experimental data suggest that amentoflavone can protect lung tissue against fibrogenic injury, at least in part, by interfering with TGF β1 signaling, leading to the downregulation of profibrotic gene expression and the suppression of extracellular matrix accumulation [29,30]. Based on this rationale, the present study was designed to investigate whether alogliptin and/or amentoflavone can mitigate BLM-induced pulmonary fibrosis in mice, and to clarify the molecular mechanisms underlying their potential protective effects.

## 2. Results

### 2.1. Comparative Effect of Different Treatments on the Lung Weight/Body Weight Index

**Figure 1:**
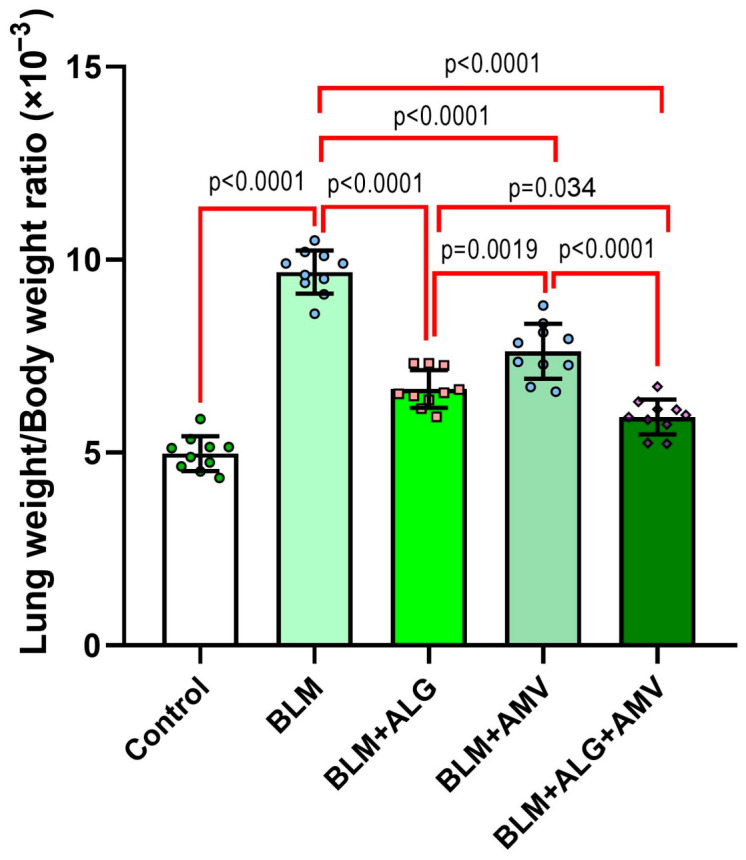
Alogliptin with or without amentoflavone ameliorated the changes in the lung weight/body weight ratio induced by bleomycin. Results are presented as the mean ± standard deviation (SD) (number of animals = 10 per group) and statistically analyzed using one-way ANOVA, followed by Tukey’s multiple comparison test. A *p*-value < 0.05 was considered significant. Abbreviations: AMV, amentoflavone; ALG, alogliptin; BLM, bleomycin.

BLM administration produced a significant elevation in the lung weight/body weight index relative to the control group. Treatment with alogliptin and/or amentoflavone markedly attenuated this increase, with the combination regimen exerting a significantly greater reduction than either monotherapy (Figure 1).

### 2.2. Comparative Effect of Different Treatments on the Total and Differential Leucocytic Count in BALF

**Figure 2:**
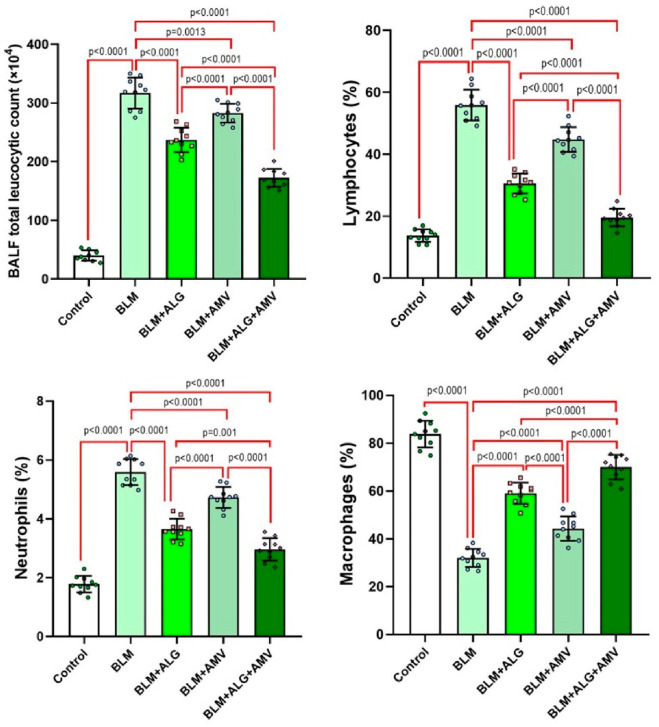
Alogliptin with or without amentoflavone mitigated the changes in BALF total and differential leucocytic counts induced by bleomycin. Results are presented as mean ± standard deviation (SD) (number of animals = 10 per group) and statistically analyzed using one-way ANOVA, followed by Tukey’s multiple comparison test. A *p*-value < 0.05 was considered significant. Abbreviations: AMV, amentoflavone; ALG, alogliptin; BALF, bronchoalveolar lavage fluid; BLM, bleomycin.

BLM administration led to a significant increase in BALF’s total leukocytic count (TLC), characterized by elevated neutrophil and lymphocyte percentages and a concomitant reduction in macrophage proportion compared with the controls. Treatment with alogliptin and/or amentoflavone significantly reduced the TLC, neutrophil, and lymphocyte fractions while restoring macrophage percentage versus the BLM group. These anti-inflammatory effects were most pronounced in the alogliptin/amentoflavone combination group compared with either agent alone (Figure 2).

### 2.3. Comparative Effect of Different Treatments on BALF LDH Activity

**Figure 3:**
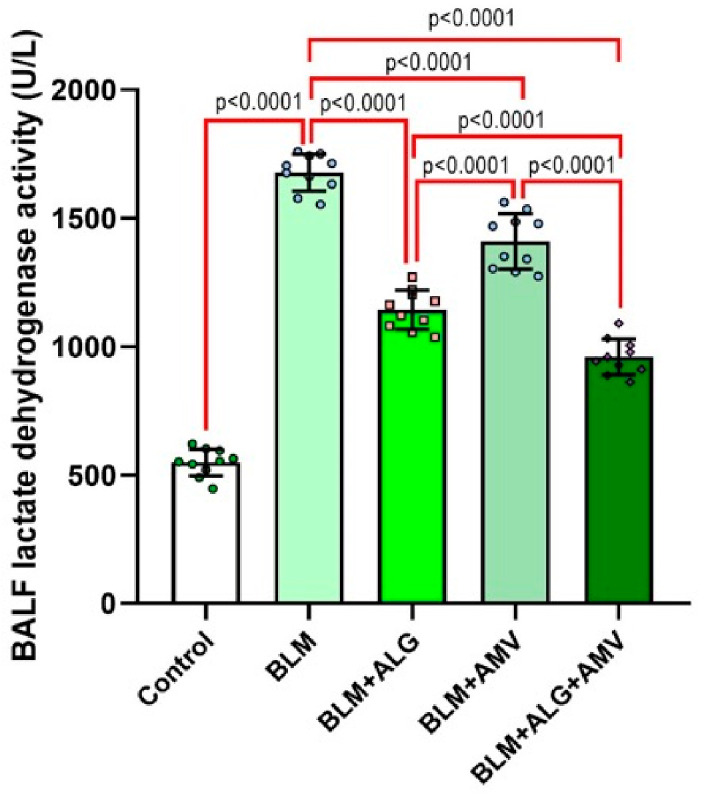
Alogliptin with or without amentoflavone reversed the changes in BALF lactate dehydrogenase activity induced by bleomycin. Results are presented as mean ± standard deviation (SD) (number of animals = 10 per group) and statistically analyzed using one-way ANOVA, followed by Tukey’s multiple comparison test. A *p*-value < 0.05 was considered significant. Abbreviations: AMV, amentoflavone; ALG, alogliptin; BLM, bleomycin; LDH, lactate dehydrogenase.

BLM administration significantly increased BALF LDH activity compared with the control group. Alogliptin and/or amentoflavone significantly reversed this elevation, with the greatest attenuation observed in the combination group compared with each monotherapy (Figure 3).

### 2.4. Comparative Effect of Different Treatments on the Lung Tissue Oxidative Stress Parameters

**Figure 4:**
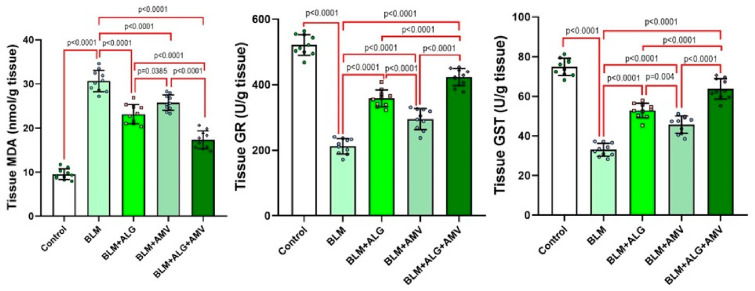
Alogliptin with or without amentoflavone ameliorated the lung tissue oxidative stress induced by bleomycin. Results are presented as mean ± standard deviation (SD) (number of animals = 10 per group) and statistically analyzed using one-way ANOVA, followed by Tukey’s multiple comparison test. A *p*-value < 0.05 was considered significant. Abbreviations: AMV, amentoflavone; ALG, alogliptin; BLM, bleomycin; GR, glutathione reductase; GST, glutathione-S-transferase; MDA, malondialdehyde.

BLM administration markedly increased the lung tissue levels of MDA and significantly reduced GR and GST activities versus the controls. Treatment with alogliptin and/or amentoflavone significantly lowered the MDA levels in the lung tissues, while restored GR and GST activities compared with the BLM group. The combined alogliptin/amentoflavone regimen produced greater improvement in the aforementioned biochemical indices than either monotherapy (Figure 4).

### 2.5. Comparative Effect of Different Treatments on Modulation of Lung Tissue SIRT1, Nrf2, and HO-1

**Figure 5:**
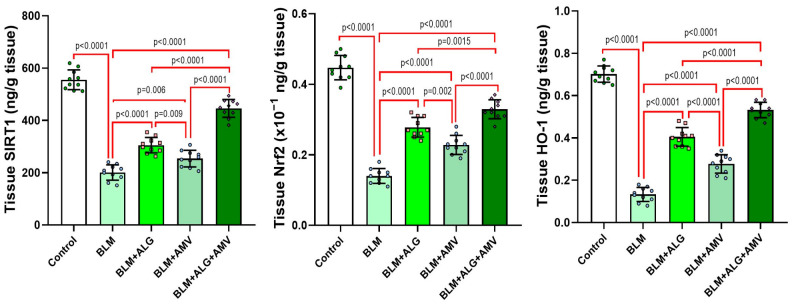
Alogliptin with or without amentoflavone ameliorated the changes in the lung tissue SIRT1, Nrf2, and HO-1 induced by bleomycin. Results are presented as mean ± standard deviation (SD) (number of animals = 10 per group) and statistically analyzed using one-way ANOVA, followed by Tukey’s multiple comparison test. A *p*-value < 0.05 was considered significant. Abbreviations: AMV, amentoflavone; ALG, alogliptin; BLM, bleomycin; HO-1, heme oxygenase-1; Nrf2, nuclear factor erythroid 2–related factor 2; SIRT1, sirtuin-1.

Intratracheal BLM significantly decreased the lung tissue SIRT1, Nrf2, and HO-1 compared with the control group. Alogliptin and/or amentoflavone significantly increased the SIRT1, Nrf2, and HO-1 levels versus the BLM group, with the most pronounced normalization in the combination group (Figure 5).

### 2.6. Comparative Effect of the Different Treatments on the Lung Tissue Proinflammatory Cytokines

**Figure 6:**
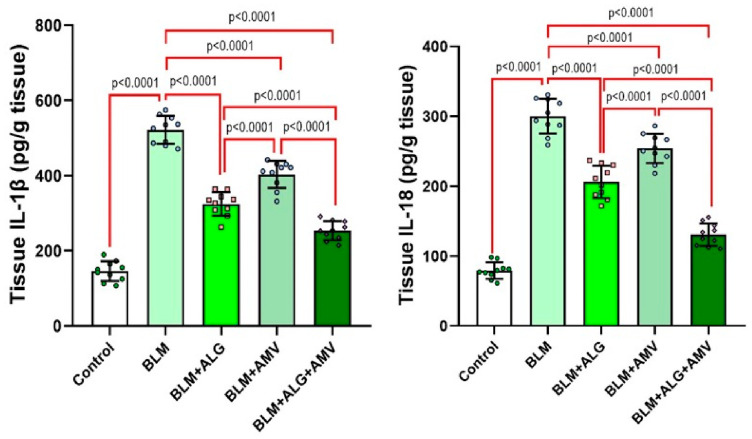
Alogliptin with or without amentoflavone ameliorated the changes in the lung tissue proinflammatory cytokines induced by bleomycin. Results are presented as mean ± standard deviation (SD) (number of animals = 10 per group) and statistically analyzed using one-way ANOVA, followed by Tukey’s multiple comparison test. A *p*-value < 0.05 was considered significant. Abbreviations: AMV, amentoflavone; ALG, alogliptin; BLM, bleomycin; IL-1β, interleukin-1-beta.

Figure 6 demonstrates that intratracheal BLM administration significantly increased the lung tissue levels of IL-1β and IL-18 when compared to the control group. Alogliptin and/or amentoflavone significantly decreased the lung tissue levels of these proinflammatory pro-pyroptotic cytokines versus the BLM group, with the most pronounced decline in the combination group.

### 2.7. Comparative Effect of the Different Treatments on the Lung Tissue TXNIP/NLRP3 Inflammasome/Gasdermin D Axis

**Figure 7:**
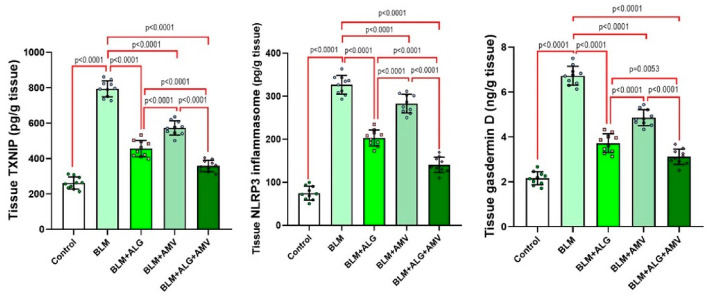
Alogliptin with or without amentoflavone combated the changes in the lung tissue TXNIP/NLRP3 inflammasome/gasdermin D axis induced by bleomycin. Results are presented as mean ± standard deviation (SD) (number of animals = 10 per group) and statistically analyzed using one-way ANOVA, followed by Tukey’s multiple comparison test. A *p*-value < 0.05 was considered significant. Abbreviations: AMV, amentoflavone; ALG, alogliptin; BLM, bleomycin; NLRP3, NOD-like receptor protein 3; TXNIP, thioredoxin-interacting protein.

As depicted in Figure 7, intratracheal BLM administration significantly increased the lung tissue levels of TXNIP, NLRP3 inflammasome, and gasdermin D relative to the control group. Alogliptin and/or amentoflavone significantly decreased the lung tissue levels of the aforementioned biochemical indices versus the BLM group, with the most significant decline being clearly evident in the combination group.

### 2.8. Comparative Effect of the Different Treatments on the Lung Tissue Levels of TGF-β1, Hydroxyproline, and Collagen

**Figure 8:**
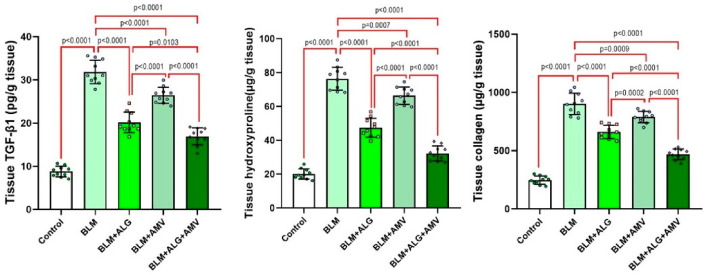
Alogliptin with or without amentoflavone mitigated the changes in the lung tissue TGF-β1, hydroxyproline, and collagen induced by bleomycin. Results are presented as mean ± standard deviation (SD) (number of animals = 10 per group) and statistically analyzed using one-way ANOVA, followed by Tukey’s multiple comparison test. A *p*-value < 0.05 was considered significant. Abbreviations: ALG, alogliptin; AMV, amentoflavone; BLM, bleomycin; TGF-β1, transforming growth factor beta 1.

Figure 8 illustrates that intratracheal BLM administration was associated with activation of the fibrogenic process, as evidenced by a significant elevation of the lung tissue levels of TGF-β1, hydroxyproline, and collagen when compared to the control animals. Alogliptin and/or amentoflavone significantly decreased the lung tissue levels of the aforementioned fibrotic indices versus the BLM group, with the most significant decline being pronounced in the combination group.

### 2.9. Comparative Effect of the Different Treatments on the Lung Tissue Levels of Beclin-1, LC3-II, and p62/SQSTM1

**Figure 9:**
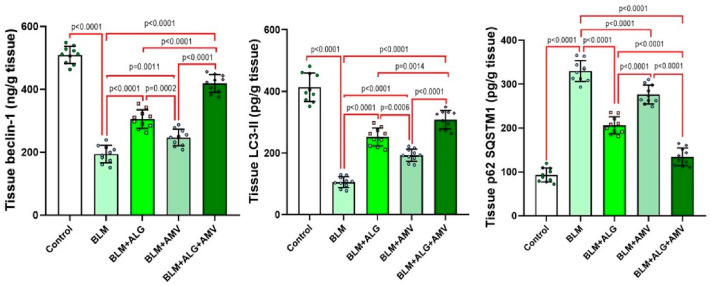
Alogliptin with or without amentoflavone ameliorated the changes in the lung tissue autophagy signals elicited by bleomycin. Results are presented as mean ± standard deviation (SD) (number of animals = 10 per group) and statistically analyzed using one-way ANOVA, followed by Tukey’s multiple comparison test. A *p*-value < 0.05 was considered significant. Abbreviations: ALG, alogliptin; AMV, amentoflavone; BLM, bleomycin; LC3-II, microtubule-associated protein 1 light chain 3; p62/SQSTM1, sequestosome 1.

The present study demonstrated that intratracheal administration of BLM was associated with significant abrogation of the autophagy process, as evidenced by a significant decline in the lung tissue levels of beclin-1 and LC3-II, associated with a significant increase in the lung tissue levels of p62/SQSTM1 when compared to the control mice. Alogliptin and/or amentoflavone significantly reversed these changes, with the most significant improvement being evidenced in the combination group (Figure 9).

### 2.10. Comparative Effect of the Different Treatments on Carbachol-Induced Contraction of the Tracheal Smooth Muscles

**Figure 10:**
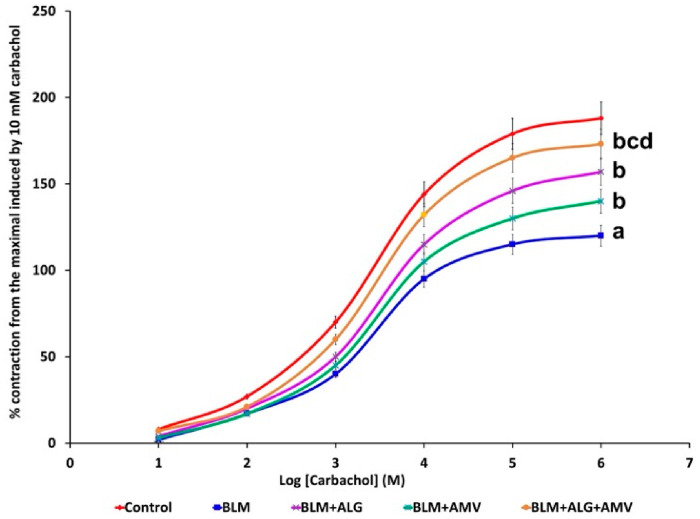
Alogliptin with or without amentoflavone ameliorated the effect of bleomycin on carbachol-induced contraction of the tracheal smooth muscles. Results are presented as mean ± standard deviation (SD) (number of animals = 10 per group) and statistically analyzed using one-way ANOVA, followed by Tukey’s multiple comparison test. A *p*-value < 0.05 was considered significant; a—significant relative to the control group, b—significant relative to the BLM-treated group, c—significant relative to the group treated with BLM + alogliptin, d—significant relative to the group treated with BLM + amentoflavone. Abbreviations: ALG, alogliptin; AMV, amentoflavone; BLM, bleomycin.

As depicted in Figure 10, BLM instillation significantly decreased carbachol-induced maximal contraction in isolated tracheal preparations compared with the control values. Administration of alogliptin and/or amentoflavone significantly restored carbachol-evoked maximal contraction relative to the BLM group, with the combination group outperforming either drug alone.

### 2.11. Comparative Effect of Alogliptin with or Without Amentoflavone on the BLM-Induced Changes in the Vascular Reactivity of Isolated Pulmonary Artery Rings

**Figure 11:**
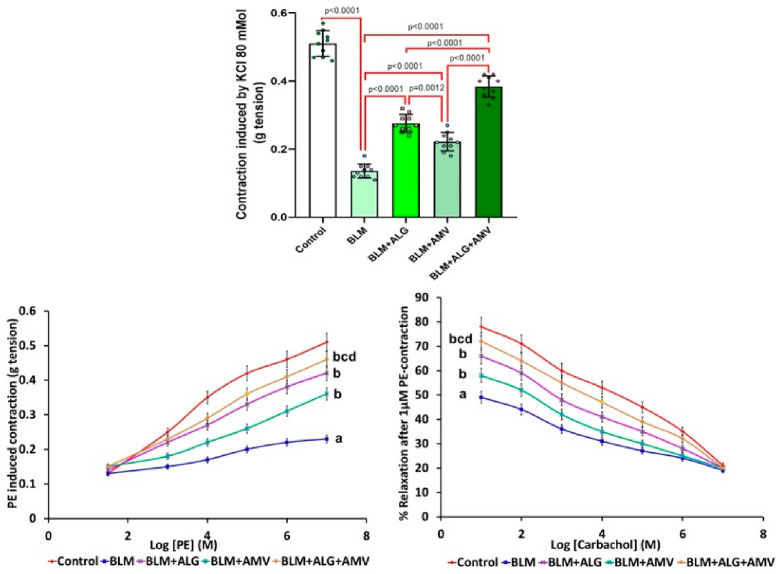
Alogliptin with or without amentoflavone mitigated the changes in the vascular reactivity of the isolated pulmonary artery rings induced by bleomycin. Results are presented as mean ± standard deviation (SD) (number of animals = 10 per group) and statistically analyzed using one-way ANOVA, followed by Tukey’s multiple comparison test. A *p*-value < 0.05 was considered significant; a—significant relative to the control group, b—significant relative to the BLM-treated group, c—significant relative to the group treated with BLM + alogliptin, d—significant relative to the group treated with BLM + amentoflavone. Abbreviations: ALG, alogliptin; AMV, amentoflavone; BLM, bleomycin; KCl, potassium chloride; PE, phenylephrine.

BLM exposure significantly reduced KCl-induced contraction, PE maximal contraction, and carbachol-induced maximal relaxation in isolated pulmonary artery rings compared with the controls. Alogliptin and/or amentoflavone significantly improved these parameters, restoring KCl and PE responses and enhancing carbachol-induced relaxation versus BLM, with superior effects for the combination therapy (Figure 11).

### 2.12. Histopathological Results

**Figure 12:**
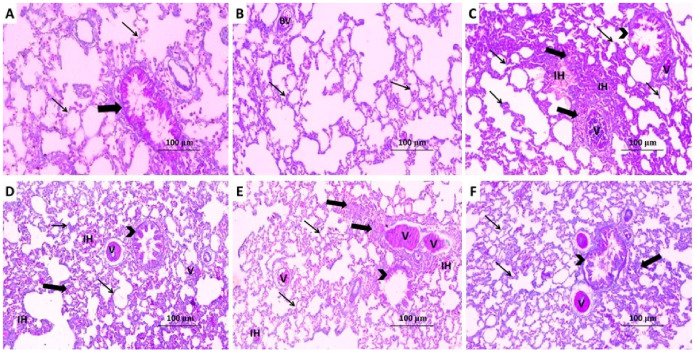
Hematoxylin and eosin stained sections (×200) of the lung tissues from (**A**,**B**) the control group showing a normal appearance of the bronchioles (thick arrow) and the alveoli with intact interalveolar septa (thin arrows) and normal appearance of the blood vessels (BV). (**C**) Bleomycin group showing massive distortion of the interalveolar septa (thin arrows), necrosis and degeneration of the bronchioles (arrow head), massive inflammatory cellular infiltration of the interstitial tissue (thick arrows), severe congestion of the pulmonary vasculature (V), with massive interstitial hemorrhage (IH). (**D**) Bleomycin group treated with alogliptin and (**E**) bleomycin group treated with amentoflavone exhibiting significant decline in the alveolar wall destruction (thin arrows), significant diminution of the congestion of the pulmonary vascular bed (V), mild infiltration with inflammatory cells (thick arrows) with restoration of the normal architecture of the bronchioles (arrow head) and mild interstitial hemorrhage (IH). (**F**) Bleomycin group treated with the alogliptin/amentoflavone combination exhibiting apparently normal alveoli (thin arrows) with minimal inflammatory cellular infiltration (thick arrow) and scanty vascular congestion (V) with restoration of the bronchiolar architecture (arrow head).

**Figure 13:**
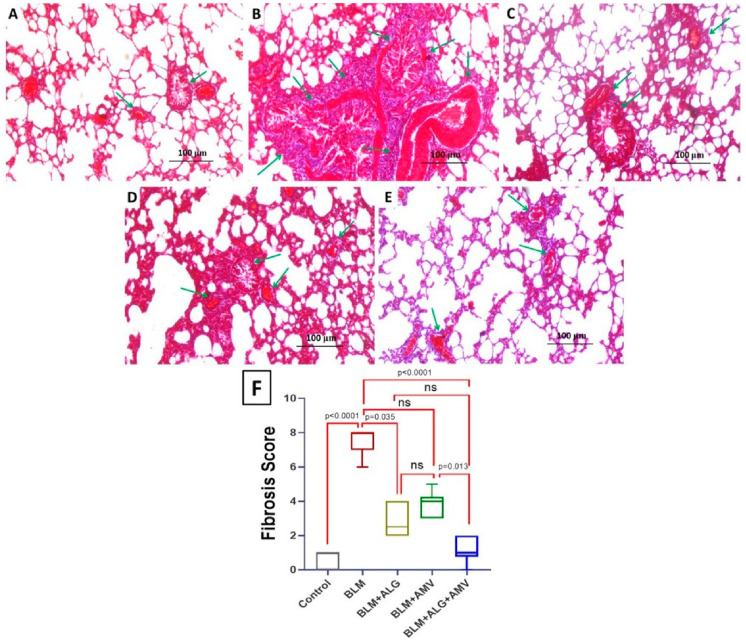
Sections of the lung stained with Mallory trichrome stain (×200) from (**A**) the control animals exhibiting minimal deposition of the collagen fibers around the bronchioles and the interstitial tissue vasculature (arrows). (**B**) Animals treated with bleomycin alone showing massive deposition of collagen fibers around the bronchioles, the alveoli, and the pulmonary blood vessels (arrows). (**C**,**D**) The animal groups that received bleomycin intratracheal instillation and treated with alogliptin or amentoflavone respectively showed a pronounced decline in the collagen fiber deposition around the pulmonary vascular bed, the bronchioles, and the alveoli (arrows). (**E**) The animal group that received bleomycin intratracheal instillation and treated with the alogliptin/amentoflavone combination showed scanty discrete deposition of the collagen fibers around the bronchioles and the pulmonary vascular bed (arrows). (**F**) Effect of alogliptin and/or amentoflavone on the fibrosis score in the bleomycin-treated mice. Values were expressed as median (interquartile range, IQR) and compared using Kruskal–Wallis followed by Dunn’s test. Number of animals = 10 mice in each group. A *p*-value less than 0.05 was considered significant. Abbreviations: ALG, alogliptin; AMV, amentoflavone; BLM, bleomycin; ns, non-significant.

The control group exhibited normal alveolar architecture with intact bronchioles, alveoli, vasculature, and interalveolar septa (Figure 12A,B) with minimal collagen fiber deposition (Figure 13A,F). BLM instillation produced marked structural derangement of the lung parenchyma, including distortion of the normal alveolar architecture, moderate to severe interstitial and intra-alveolar hemorrhage, emphysema-like airspace enlargement, pronounced thickening of the interalveolar septa, and dense leukocytic infiltration, accompanied by a significant elevation in the fibrosis score versus the controls (Figure 12C and Figure 13B,F). In contrast, treatment with either alogliptin or amentoflavone significantly attenuated the inflammatory and fibrotic changes, with noticeable restoration of alveolar architecture, reduction in septal thickening, hemorrhage, and emphysematous areas (Figure 12D,E), and a significant decrease in the fibrous tissue deposition and the fibrosis score relative to the BLM group (Figure 13C,D,F). The most pronounced histological protection was observed in the alogliptin/amentoflavone combination group, which showed near-normalization of the lung tissue structure (Figure 12F) and the greatest reduction in the fibrosis score compared with either monotherapy (Figure 13E,F).

### 2.13. Immunohistochemical Results

**Figure 14:**
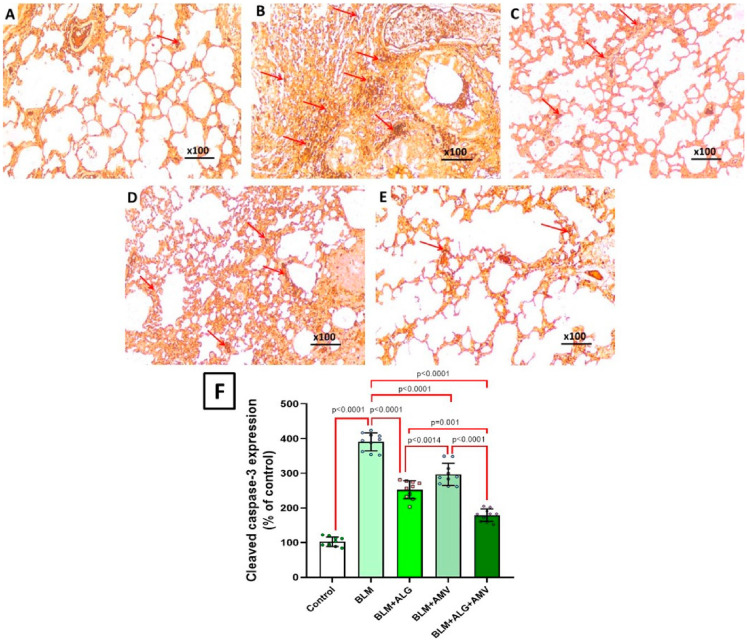
A photomicrograph demonstrating the immunohistochemical staining of the lung tissue specimens for cleaved caspase-3 (×200) from (**A**) the control group showed minimal positive immune expression of cleaved caspase-3 (arrow); (**B**) the group that received a single intratracheal instillation of bleomycin showed strong positive immune expression of cleaved caspase-3 (arrows); (**C**) the bleomycin group treated with alogliptin and (**D**) the bleomycin group treated with amentoflavone showed moderate positive immune expression of cleaved caspase-3 (arrows); (**E**) the bleomycin group treated with alogliptin/amentoflavone combination showing mild positive immune expression of cleaved caspase-3 (arrows); and (**F**) The percentage effect of the different treatments on the immune expression of cleaved caspase-3 in the lung tissues of bleomycin-treated mice. Values are expressed as mean ± standard deviation. Number of animals = 10 mice per group. One-way analysis of variance (ANOVA) was used to assess the differences between the different groups, and then Tukey’s multiple comparisons post-hoc test was employed. A *p*-value less than 0.05 was considered significant. Abbreviations: ALG, alogliptin; AMV, amentoflavone; BLM: bleomycin.

**Figure 15:**
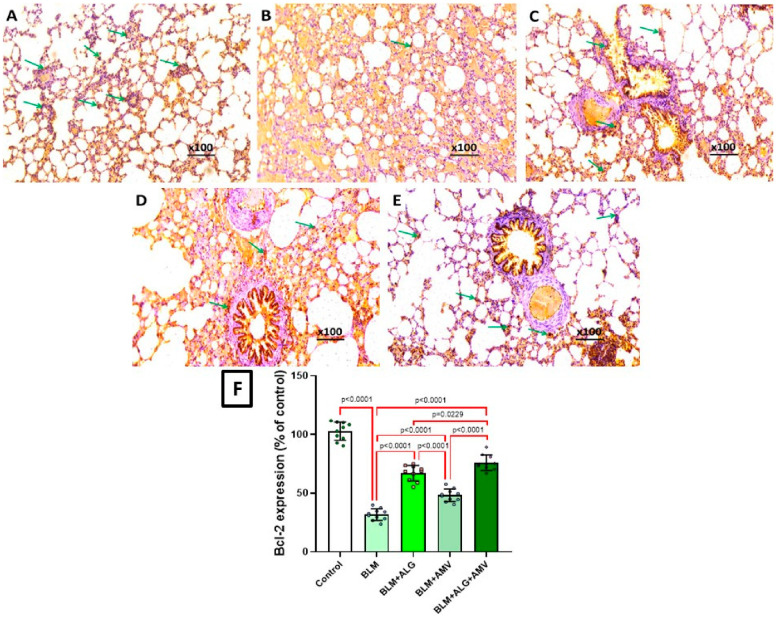
A photomicrograph demonstrating the immunohistochemical expression of Bcl-2 in the lung tissue specimens (×200) from (**A**) the control group showed strong positive immune expression of Bcl-2 (arrows); (**B**) the group that received a single intratracheal instillation of bleomycin showed minimal positive immune expression of Bcl-2 (arrow); (**C**) the bleomycin group treated with alogliptin and (**D**) the bleomycin group treated with amentoflavone showed moderate positive immune expression of cleaved caspase-3 (arrows); (**E**) the bleomycin group treated with alogliptin/amentoflavone combination showed strong positive immune expression of Bcl-2 (Arrows); and (**F**) the percentage effect of the different treatments on the immune expression of Bcl-2 in the lung tissue specimens of bleomycin-treated animals. Values are expressed as mean ± standard deviation. Number of animals = 10 mice per group. One-way analysis of variance (ANOVA) was used to assess the differences between the different groups, and then Tukey’s multiple comparisons post hoc test was employed. A *p*-value less than 0.05 was considered significant. Abbreviations: ALG, alogliptin; AMV, amentoflavone; Bcl-2, beta cell lymphoma-2; BLM: bleomycin.

BLM instillation was associated with a marked activation of the apoptotic pathways in the lung tissues, evidenced by a significant rise in tissue cleaved caspase-3 immune expression (Figure 14B) associated with a significant decline in tissue Bcl-2 immunostaining (Figure 15B) compared with the control group. Conversely, treatment with alogliptin and/or amentoflavone significantly exhibited potent antiapoptotic effects in lung tissue, as evidenced by a marked decrease in cleaved caspase-3 immunostaining (Figure 14C,D) and a significant increase in Bcl-2 immunostaining (Figure 15C,D) compared to the BLM group. The greatest decline in the lung tissue cleaved caspase-3 positivity and the most pronounced increase in the lung tissue Bcl-2 immunostaining were observed in the alogliptin/amentoflavone combination group (Figure 14E and Figure 15E, respectively), which outperformed either monotherapy.

## 3. Discussion

The current study elucidated the therapeutic potential of alogliptin and amentoflavone in BLM-induced pulmonary fibrosis and offers insights into the underlying mechanisms involved. BLM-induced pulmonary fibrosis was induced through a single intratracheal instillation, a well-validated rodent model that reproduces the sequential phases of epithelial injury, inflammation, fibrogenesis, and functional decline seen in human disease [31]. Consistent with previous reports, BLM administration provoked marked biochemical derangements, disruption of lung architecture, collagen accumulation, and the deterioration of pulmonary mechanics and gas exchange [32]. Mechanistically, the model illustrates the established pathophysiology in which BLM induces prolonged oxidative stress and inflammation that exacerbate each other, disrupting normal injury and repair mechanisms, ultimately resulting in progressive fibrotic remodeling [33]. BLM binds to ferrous ions and undergoes redox cycling, generating ROS that induce DNA strand breaks and oxidatively damage proteins and membrane lipids, driving lipid peroxidation and cell death, which in turn initiates inflammatory and profibrotic cascades [34]. These events reinforce the biochemical, histological, and physiological abnormalities observed in the present BLM lung fibrosis model.

In the present study, the observed reduction in macrophages within BALF following BLM administration should not be interpreted simply as a numerical decline. Rather, it reflects a dynamic process that involves the depletion of the resident alveolar macrophages, migration of subsets, and functional polarization [35]. Early after injury, alveolar macrophages undergo apoptosis, leading to an apparent reduction in their total counts. Subsequently, monocyte-derived macrophages are recruited and undergo polarization toward pro-fibrotic phenotypes, which contribute to extracellular matrix deposition and sustained inflammation. This shift in macrophage populations is a critical driver of fibrosis progression [36]. While we quantified the total macrophage number in the current study, the findings align with this established paradigm, suggesting that the alogliptin/amentoflavone combination may modulate macrophage dynamics in addition to reducing neutrophil and lymphocyte infiltration. By influencing both innate and adaptive immune responses, an alogliptin/amentoflavone combination may restore the balance between the inflammatory and reparative processes, thereby mitigating BLM-induced fibrotic remodeling.

The production of ROS caused by oxidative stress is a major cause of BLM-induced pulmonary fibrosis, and restoring redox balance is a key way to combat fibrosis [3]. BLM-induced fibrosis is closely linked to increased ROS and lipid peroxidation, characterized by elevated MDA levels and depletion of the enzymatic antioxidants [37]. In the present study, intratracheal BLM instillation exhibited redox imbalance, as evidenced by a marked increase in lung MDA levels accompanied by a significant reduction in GR and GST activities. Given this pathogenic role, compounds capable of suppressing ROS generation, limiting lipid peroxidation, and reinforcing endogenous antioxidant defenses are widely regarded as promising candidates for antifibrotic intervention [38]. Consistent with this concept, treatment with alogliptin and/or amentoflavone markedly ameliorated BLM-induced oxidative perturbations, normalizing MDA and restoring antioxidant enzyme activities. The protection against BLM-induced oxidative lung injury is probably due to stabilizing membrane phospholipids, reducing lipid peroxidation products, and strengthening the antioxidant system [24,39].

Bleomycin-induced pulmonary fibrosis is closely linked to suppression of the SIRT1/Nrf2/HO-1 signaling axis, which normally functions as a master regulator of oxidative stress, inflammation, and tissue remodeling [40]. SIRT1, a NAD^+^-dependent deacetylase, enhances the activity of Nrf2, a transcription factor that governs antioxidant defense mechanisms [41]. Once activated, Nrf2 promotes the expression of HO-1, an enzyme with well-proven strong cytoprotective and anti-inflammatory properties in the pulmonary tissues [42]. Together, this signaling cascade reduces ROS, limits fibroblast activation, and mitigates extracellular matrix deposition, thereby slowing the progression of fibrotic remodeling in the lungs [40]. BLM disrupts this pathway, resulting in excessive ROS generation, cellular senescence, and uncontrolled collagen deposition [43]. The comparative effect of different treatments on modulating SIRT1, Nrf2, and HO-1 is therefore crucial, as restoration of this axis can rebalance redox homeostasis, attenuate inflammatory signaling, and reduce fibrotic remodeling. Consistent with this concept, intratracheal BLM administration in the current study was associated with a significant decline in the SIRT1, Nrf2, and HO-1 content of the pulmonary tissues relative to the control group. Notably, treatment with alogliptin and/or amentoflavone significantly increased the lung SIRT1, Nrf2, and HO-1 contents relative to the BLM group. This reduction may be interpreted as a consequence of their antioxidant actions by attenuating BLM-induced ROS generation, and hence augmenting the antioxidant potential of both alogliptin and amentoflavone [25,39]. These findings highlight the importance of targeting the SIRT1/Nrf2/HO-1 axis as a strategy to mitigate fibrosis progression and support the translational relevance of our results.

As evidenced in the current study, the TXNIP/NLRP3 inflammasome/gasdermin D axis is a critical pathway that may effectively contribute to the development of bleomycin-induced lung fibrosis [9,13]. TXNIP acts as a sensor of oxidative stress and facilitates the activation of the NLRP3 inflammasome, a multiprotein complex that drives inflammatory signaling [44]. Once activated, the NLRP3 inflammasome activates caspase-1, which in turn cleaves gasdermin D, which forms pores in the cell membrane, promoting pyroptotic cell death and the release of pro-inflammatory mediators [45]. Additionally, this cascade promotes fibroblast activation, ultimately leading to excessive deposition of extracellular matrix and fibrotic remodeling in the lungs [46].

A key consequence of the TXNIP/NLRP3 inflammasome/gasdermin D axis is the maturation and secretion of IL-1β and IL-18, which fuel the inflammatory environment in bleomycin-induced fibrosis [13]. IL-1β enhances the recruitment of immune cells and stimulates fibroblast proliferation, while IL-18 amplifies the inflammatory signaling and tissue damage [47]. As depicted in the present study, these cytokines perpetuate a cycle of inflammation and fibrosis, worsening the pulmonary functions [48]. By linking oxidative stress to inflammasome activation and cytokine release, the TXNIP/NLRP3/gasdermin D axis serves as a central driver of the inflammatory and fibrotic responses in bleomycin-injured lungs [49].

In the present study, alogliptin treatment significantly reduced the expression of TXNIP, NLRP3 inflammasome, gasdermin D, IL-1β, and IL-18 compared with the BLM group. These findings align with evidence indicating that DPP 4 inhibitors exert potent anti-inflammatory and anti-pyroptotic actions independent of their glucose-lowering properties [50]. Similarly, amentoflavone in the current study produced a significant abrogation of the TXNIP/NLRP3/gasdermin D axis and was associated with a significant decline in IL-1β, and IL-18 levels in the lung tissues relative to the BLM group, aligning with prior reports that amentoflavone possesses a strong anti-pyroptotic property via inhibition of the NLRP3 inflammasome and its downstream signals, which is also reflected on the pro inflammatory pathways [21].

The ongoing research has proven that TGF-β1 is a central mediator in bleomycin-induced lung fibrosis [51]. This role may be through the activation of fibroblasts and their transformation into myofibroblasts, which are responsible for excessive collagen deposition and lung tissue scarring [52]. Hydroxyproline serves as a biochemical marker of collagen content, reflecting the extent of fibrotic remodeling within the lung tissue. Elevated levels of hydroxyproline indicate increased collagen synthesis and deposition, directly correlating with the severity of fibrosis [53]. In agreement with the results of the current study, TGF-β1 and hydroxyproline highlight both the mechanistic drivers and measurable outcomes of bleomycin-induced lung fibrosis, linking molecular signaling with structural damage [54]. Administration of alogliptin and/or amentoflavone in the present study significantly reduced the TGF β1, hydroxyproline, and collagen contents of the lung compared with the BLM group, suggesting that downregulation of TGF β1 and its downstream mediators represents an additional mechanism underlying the anti-fibrotic actions of both agents [15,55].

Autophagy functions as a protective mechanism, clearing damaged organelles and reducing oxidative stress, thereby limiting cellular injury and fibrotic signaling in the pulmonary tissues [56]. In contrast, excessive apoptosis leads to the loss of alveolar epithelial cells, disrupting tissue integrity and promoting fibroblast activation and collagen deposition [57]. When autophagy is insufficient, apoptosis predominates, fueling inflammation and fibrotic remodeling [58]. This was clearly evidenced in the present study where intratracheal instillation of bleomycin was associated with a significant increase in cleaved caspase-3 and p62/SQSTM1 expression, together with a significant decline in Bcl-2, beclin-1, and LC3-II expression in the lung tissues relative to the control animals. Conversely, treatment with alogliptin and/or amentoflavone in the present study maintained a healthy balance between these processes, which helped to preserve lung structure and reduce the severity of fibrosis, highlighting the importance of apoptosis/autophagy interplay in determining disease outcomes [15,25].

In the present study, BLM instillation, aligned with prior findings of biochemical and histopathological injury, was correlated with considerable vascular and tracheal dysfunction. BLM significantly diminished the vascular reactivity of isolated pulmonary artery rings to KCl, PE, and carbachol. This was in line with Zaghloul et al. [59], who attributed these effects to BLM-elicited impairment of the vasoconstrictor responsiveness. Likewise, in vitro assessment of tracheal smooth muscles revealed a significant decrease in contractile responses to carbachol. This agrees with Qin et al. [60] who reported that these effects represent direct consequences to BLM-mediated impairment of the functions of airway smooth muscles. Conversely, treatment with alogliptin and/or amentoflavone in the current work effectively reversed these BLM-induced functional disturbances. These findings functionally reinforce the aforementioned biochemical and histopathological improvements achieved with alogliptin and/or amentoflavone administration. These results highlight the translational relevance of targeting the vascular, parenchymal, and airway components in pulmonary fibrosis.

In the current study, the alogliptin/amentoflavone combination produced a greater protective effect than either agent alone. The rationale for combining alogliptin and amentoflavone may stem from their complementary pharmacological actions. Alogliptin, a clinically approved DPP-4 inhibitor, has demonstrated anti-inflammatory and anti-fibrotic effects beyond glycemic control, partly through the modulation of oxidative stress and apoptotic pathways. Amentoflavone, a naturally occurring biflavonoid, is recognized for its potent antioxidant and anti-inflammatory properties, with reported efficacy in models of fibrosis. Although no previous studies have directly examined the alogliptin/amentoflavone combination in bleomycin-induced lung fibrosis, the individual protective effects of each agent against oxidative stress and tissue injury provided the scientific basis for exploring their synergistic potential. These findings may align with previous reports that suggest that the combination of DPP 4 inhibitors and flavonoids can exert beneficial tissue protective effects irrespective of the glycemic control [61]. This may be attributed to the DPP-4 inhibitory capacity of amentoflavone, which may add a synergistic effect to alogliptin [62]. In addition, amentoflavone itself inhibits the CYP3A4 enzyme, which is the primary metabolic route for alogliptin, thereby increasing its bioavailability and efficacy [63,64].

The recent research efforts have proven that alogliptin has a well-established safety profile in humans, with adverse effects generally mild and infrequent [65], while amentoflavone has been widely studied as a dietary flavonoid with low toxicity in preclinical models [24]. To date, no significant drug–drug interactions between alogliptin and flavonoids have been reported. Nevertheless, further pharmacokinetic and toxicological investigations are warranted to fully establish the safety of this combination in clinical settings.

Although the present study yielded encouraging results, several limitations must be considered. The small sample size, along with inherent differences between mice and humans regarding metabolism and immunity, may restrict how broadly the findings of the current work can be applied. In addition, the investigations performed did not assess the potential adverse effects of alogliptin or amentoflavone. Furthermore, the present study did not explore whether these agents may alter the anticancer activity of bleomycin, an important factor for clinical relevance. Furthermore, mechanistic understanding was limited by reliance on ELISA and immunohistochemistry, without additional validation through advanced techniques such as Western blotting, mRNA expression profiling, or knockout animal models.

## 4. Materials and Methods

### 4.1. Drugs and Chemicals

Bleomycin sulfate was provided by Cayman Chemical (Ann Arbor, MI 48108, USA; CAS number 9041-93-4, Item number 13877, purity ≥95%). Alogliptin, amentoflavone, and carboxymethylcellulose (CMC) were purchased from ChemSrc (Shanghai, China; CAS numbers 850649-61-5, 1617-53-4, and 9004-32-4, respectively; purity 98.0%). Bleomycin was dissolved in 0.9% sodium chloride solution. Both alogliptin and amentoflavone were suspended in 0.5% CMC solution. The other drugs, chemicals, and reagents used in the present study were obtained from Sigma Aldrich Chemical Co. (St. Louis, MO 63178, USA).

### 4.2. Experimental Animals

In this experiment, fifty male C57BL/6 mice (20–28 g) were obtained from the animal house of the Faculty of Science, Tanta University, Egypt. Animals were allowed a 2-week acclimatization period before the onset of the experimental procedures. Mice were housed in a dedicated room under controlled environmental conditions (25 ± 3 °C, 60 ± 10% relative humidity) with 12 h light/dark cycles and ad libitum access to standard chow and tap water. All of the methods and animal handling in the present study were performed in accordance with the ARRIVE guidelines, the U.K. Animals (Scientific Procedures) Act of 1986, and the EU Directive 2010/63/EU governing animal experimentation. The study protocol attained official approval from the Research Ethics Committee of the Faculty of Medicine, Tanta University, Tanta, Egypt (Approval ID: 36264PR131011016/2/26). Animal grouping and randomization were carried out using a computer-generated randomization process, which utilized the RAND function in Microsoft Excel. Also, the persons who were responsible for animal care, harvesting biological specimens, biochemical assessment, and histopathological examination were totally blinded to the experimental protocol and animal grouping to verify complete impartiality and decrease the chance for observer bias.

### 4.3. Experimental Protocol

#### 4.3.1. Induction of Pulmonary Fibrosis

Pulmonary fibrosis was induced by a single intratracheal instillation of bleomycin (BLM, 2.5 mg/kg) prepared as the sulfate salt in 0.1 mL of sterile 0.9% saline solution, administered under thiopental sodium (60 mg/kg, intraperitoneally) anesthesia, thereby minimizing distress to the animals. A midline cervical incision was performed to expose the trachea, and the BLM solution was carefully instilled into the tracheal lumen. Following instillation, animals were maintained in a vertical position and gently rotated several times to facilitate a homogeneous distribution of BLM throughout the lung parenchyma, as commonly described in intratracheal bleomycin models. To close the incision, standard surgical suturing methods were used. To reduce the risk of local infection, a 2% sodium fusidate preparation was then put on the wound [26].

#### 4.3.2. Animals Grouping

**Figure 16:**
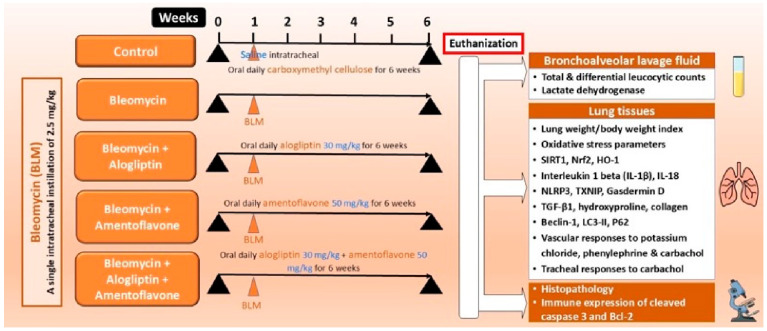
Animal grouping and the experimental design of the present study. Abbreviations: Bcl-2, beta cell lymphoma-2; BLM, bleomycin; HO-1, heme oxygenase-1; IL-1β, interleukin-1 beta; LC3-II, microtubule-associated protein 1 light chain 3; NLRP3, NOD-like receptor family pyrin domain-containing; Nrf2, nuclear factor erythroid 2–related factor 2; P62, sequestosome 1; SIRT1, sirtuin-1; TGF-β1, transforming growth factor beta 1, TXNIP, thioredoxin-interacting protein.

Mice were randomly divided into five equal groups (10 mice/group), as demonstrated in Figure 16 as follows: a control group in which mice received a single intratracheal instillation of 0.1 mL of 0.9% saline solution together with daily oral administration of 1% CMC solution by oral gavage starting one week before and continuing for 5 weeks after BLM instillation; the BLM group in which mice received BLM (2.5 mg/kg) via intratracheal instillation as described above; the alogliptin + BLM group in which mice were treated with alogliptin (30 mg/kg/day) by oral gavage once daily, starting one week before and continuing for 5 weeks after BLM instillation for a total of six weeks of alogliptin administration [27]; the amentoflavone + BLM group in which mice were treated with amentoflavone (50 mg/kg/day) by oral gavage once daily, starting one week before and continuing for 5 weeks after BLM instillation for a total of six weeks of amentoflavone administration [28]; and the alogliptin + amentoflavone + BLM group in which BLM-treated mice were given alogliptin concurrently with amentoflavone by oral gavage, at the same doses described above for the duration of the experiment.

At the end of five weeks after BLM instillation, mice were weighed and anesthetized with thiopental sodium (60 mg/kg body weight, intraperitoneal). The thoracic cavity was opened, and the tracheas were exposed to collect bronchoalveolar lavage fluid (BALF) and for excision of the trachea and pulmonary artery for in vitro assays. The lungs were isolated, rinsed in ice-cold saline, and weighed to calculate the lung weight-to-body weight index. Parts of the left lung were homogenized using a fully automatic ultrasonic extraction for ultrasonic emulsifier homogenizer tissue breaking (Ningbo Movel Scientific Instrument Co., Ltd., Ningbo, Zhejiang, China). For biochemical analyses, the tissue homogenate was centrifuged at 1008× *g* for 15 min at 4 °C using a Medifuge™ Centrifuge (Thermo Fisher Scientific Inc., Waltham, MA 02451, USA; catalog # 75008800), where the yielded supernatant was the working solution upon which the biochemical assays were performed. Other portions of the lobes of the left lung were reserved for histopathological and immunohistochemical evaluation.

### 4.4. Harvesting the Bronchoalveolar Lavage Fluid (BALF)

The thoracic cavity was opened, the trachea was exposed and cannulated, and bronchoalveolar lavage was performed by gently instilling 2 mL of sterile 0.9% saline into the lungs three consecutive times. Per lavage, about 50–70% of the instilled volume was recovered, which is consistent with reported murine BAL recovery rates. Recovery of lavage fluid was optimized by gently compressing the chest wall several times during aspiration, as commonly recommended to maximize the BALF yield while minimizing mechanical stress on the airways. The collected BALF fractions were centrifuged at 2000 rpm, 4 °C for 10 min using a high-speed cooling centrifuge (Model IG-243R, iGene Labserve Pvt. Ltd., New Delhi, India). The resulting cell pellets were combined and resuspended in 500 µL of sterile saline to prepare a uniform cell suspension for the determination of total and differential leukocytic counts.

### 4.5. Quantification of BALF Total and Differential Leucocytic Counts

The total and differential leucocytic counts in BALF were quantified using a hemocytometer (Marienfeld, nebular improved, Paul Marienfeld GmbH & Co. KG, Lauda-Koenigshofen, Germany) after staining with Turk solution, which was prepared as a mixture of 3 mL glacial acetic acid, 1mL gentian violet 1%, and then made to a volume of 100 mL by distilled water [29].

### 4.6. Evaluation of BALF Lactate Dehydrogenase (LDH) Activity

LDH activity was quantified using commercially available assay kits, following the respective manufacturers’ protocols, as is standard in experimental and clinical studies (BioAb Co., Ltd., New Taipei City, Taiwan; catalog # RK02980).

### 4.7. Assessment of the Lung Tissue Oxidative Stress Parameters

The lung tissue malondialdehyde (MDA) levels were assayed according to Senthilkumar et al. [30], in which MDA reacts with thiobarbituric acid (TBA), forming a colored adduct that absorbs light at a wavelength of 532 nm. The activities of glutathione reductase (GR) and glutathione-S-transferase (GST) were quantified using sandwich ELISA kits (ELK Biotechnology CO., Ltd., Sugar Land, TX 77478, USA; catalog # ELK5010 and ELK9346, respectively) according to the vendor’s instructions.

### 4.8. Determination of the Lung Tissue Sirtuin-1 (SIRT1), Nuclear Factor Erythroid 2-Related Factor 2 (Nrf2), and Heme Oxygenase-1 (HO-1)

The lung tissue content of SIRT1 was quantified using sandwich ELISA kits according to the vendor’s instructions (LSBio, Newark, CA 94560, USA; catalog # LS-F53396). The lung tissue content of Nrf2 and HO-1 was determined using ELISA kits purchased from Novus Biologicals (Novus Biologicals, part of Bio-Techne, Minneapolis, MN 55413, USA; catalog # NBP2-76758 and NBP3-18029, respectively) following the supplier’s instructions.

### 4.9. Quantification of the Lung Tissue Content of the Pro-Inflammatory Pro-Pyroptotic Cytokines

Biomatik Corporation was the source of the sandwich ELISA kits used for the assessment of the levels of interleukin 1 beta (IL-1β) and IL-18 according to the enclosed instructions (Biomatik Corporation, Kitchener, ON, N2C 1N6, Canada; catalog # EKF57763 and EKC37154, respectively).

### 4.10. Assessment of the TXNIP/NLRP3 Inflammasome/Gasdermin D Axis in the Lung Tissues

Biorbyt provided the ELISA kits used for assessment of the lung tissue levels of TXNIP and NLRP3 inflammasome according to the provider’s guide (Biorbyt Ltd., Durham, NC 27713, USA; catalog # orb408689 and orb692916, respectively). The levels of gasdermin D in the lung tissues were quantified following the instructions of sandwich ELISA kits supplied by Biomatik (Biomatik Corporation, Kitchener, ON, N2C 1N6, Canada; catalog # EKU09198).

### 4.11. Determination of TGF-β1, Hydroxyproline, and Collagen Content of the Lung Tissues

Cusabio was the source of the ELISA kits used for the assessment of levels in the lung tissues according to the enclosed data sheet (Cusabio, Jiangxia District, Wuhan, Hubei Province, China; catalog # CSB-E04726m). The lung tissue content of hydroxyproline was quantified using ELISA kits purchased from AFG Bioscience (AFG Bioscience, Northbrook, IL 60062, USA; catalog # EK730185). The lung tissue content of collagen was calculated from the following established equation (Lung collagen content = hydroxyproline content × 13.5).

### 4.12. Assessment of the Autophagic Signals in the Lung Tissues of the Different Groups

The lung tissue content of beclin-1 was assessed using ELISA kits supplied by FineTest (FineTest, Wuhan, 430074, Hubei, China; catalog # EM0492). Cell Biolabs provided the ELISA kits used for the determination of the lung tissue levels of microtubule-associated protein 1 light chain 3 (LC3-II) (Cell Biolabs, Inc., San Diego, CA 92111, USA; catalog # CBA-5116). The anti-autophagy marker sequestosome 1 (p62/SQSTM1) levels were quantified in the lung tissues using ELISA kits supplied by Novus Biologicals (Novus Biologicals, part of Bio-Techne, Minneapolis, MN 55413, USA; catalog # NBP2-61300). The aforementioned parameters were assayed according to the providers’ instructions.

### 4.13. In Vitro Determination of Reactivity of the Pulmonary Arteries to Potassium Chloride (KCl), Phenylephrine (PE) (10^−9^–10^−5^ M), and Carbachol (10^−8^–10^−5^ M)

The first branch of the main pulmonary artery was rapidly excised and immersed in cold oxygenated Krebs–Henseleit solution. The vessels were carefully cleaned of adherent fat and connective tissue and cut into ring segments (2–4 mm in length), which were mounted in 10 mL organ baths maintained at 37 °C and aerated with 95% O_2_/5% CO_2_. Rings were set at a resting tension of 0.8 g and allowed to equilibrate for 60 min. Isometric tension generated by the vascular pulmonary artery rings was measured using a K30 force transducer (WIKA Alexander Wiegand SE & Co. KG, 63911 Klingenberg, Germany) and recorded with a Power lab unit/400 linked to a PC running Chart v4.2 software (PassMark Software, Surry Hills, NSW 2010, Australia). After equilibration, vascular responsiveness was assessed. Contractile capacity was first verified with 80 mmol/L KCl, and responses to semi logarithmic concentration of phenylephrine (PE) (10^−9^–10^−5^ M) were then constructed and expressed as g tension; from these, the maximal effect (Emax) and the concentration producing 50% of Emax (EC_50_) were determined, and pD_2_ values (−log_10_EC_50_) were calculated by constructing cumulative concentration–response curves. Endothelium-dependent relaxation was evaluated in response to carbachol (10^−8^–10^−5^ M) in rings precontracted with PE (1 µM), as commonly applied in pulmonary artery vasoreactivity studies [66].

### 4.14. In Vitro Determination of the Reactivity of the Tracheal Smooth Muscles to Carbachol (10^−9^–10^−4^ M)

The tracheae were excised and transferred to Krebs–Henseleit solution (KHS) gassed with a carbogen mixture (95% O_2_/5% CO_2_). After the removal of adhering connective tissue, tracheal strips were prepared in a zigzag configuration and mounted in organ baths at 37 °C and bubbled with carbogen under 1 g resting tension. Preparations were allowed to stabilize for 60 min, with the KHS solution renewed every 15 min. Isometric tension was measured using a K30 force transducer (WIKA Alexander Wiegand SE & Co. KG, 63911 Klingenberg, Germany) and recorded via PowerLab/Chart (PassMark Software, Surry Hills, NSW 2010, Australia) as for the pulmonary rings. To verify contractile integrity, tracheal preparations were initially challenged with carbachol (10^−4^ M) to obtain a maximal contraction, then washed repeatedly with fresh KHS until baseline tension was restored. Cumulative concentration–response curves to carbachol (10^−9^–10^−4^ M) were then generated. From individual curves, Emax and EC_50_ were calculated, and results were expressed as a percentage of the maximal contraction induced by 10^−4^ M carbachol, consistent with the established tracheal smooth muscle methodology [67].

### 4.15. Light Microscopic Examination of the Histopathological Changes of the Lung Tissues

The left upper lung lobes were excised, rinsed in ice-cold saline, and fixed in 10% neutral-buffered formalin for 24 h. Samples were then processed for paraffin embedding, sectioned, and stained with hematoxylin and eosin to assess alveolar and interstitial injury, and with Mallory trichrome to visualize collagen deposition and extracellular matrix accumulation, following standard pulmonary histopathology practice. Sections were examined by light microscopy in a random order by a pathologist blinded to group allocation. Lung fibrosis was scored according to the modified Ashcroft method described by Hübner et al. [68] using a semiquantitative 0–8 scale as follows: 0 denotes normal lung; 1, isolated alveolar septal thickening with mild fibrosis; 2, septal fibrosis with knot-like lesions; 3, contiguous fibrotic thickening of alveolar septa; 4, single discrete fibrotic masses; 5, confluent fibrotic masses; 6, extensive, contiguous fibrotic areas; 7, cystic change with air-space enlargement; and 8, complete fibrous obliteration of lung architecture.

### 4.16. Immunohistochemical Evaluation of Cleaved Caspase-3 and Beta Cell Lymphoma-2 (Bcl-2) Expression in the Lung Tissue Specimens

This was performed according to Buyuklu et al. [69] and Groeger et al. [70], respectively. Briefly, the formalin-fixed, paraffin-embedded lung sections were deparaffinized, rehydrated through graded ethanols, and incubated with 5% bovine serum albumin (BSA) in Tris-buffered saline (TBS) for 2 h to block non-specific binding. Slides were incubated overnight at 4 °C with a mouse monoclonal antibody targeting cleaved caspase-3 (Abcam, Waltham, MA, USA; clone # 31A1067) and a mouse monoclonal antibody against Bcl-2 sourced from Cell Signaling Technology (Boston, MA, USA; catalog # 15071). Following washes, a biotinylated secondary antibody (MyBioSource, San Diego, CA, USA; catalog # MBS9610384) was used to detect the caspase-3 primary antibody, while a horseradish peroxidase-conjugated secondary antibody (St John’s Laboratory Ltd., London, UK; catalog # STJA0041594) was applied for Bcl-2. Signal development employed the avidin–biotin complex method for caspase-3 and a DAB chromogen to visualize Bcl-2 as a brown precipitate. Hematoxylin served as a nuclear counterstain, after which slides were dehydrated through graded ethanol, cleared in xylene, and mounted with Permount and coverslips. Staining intensity was assessed using a Leica DM2000 light microscope (Leica Microsystems, Wetzlar, Germany) across ten randomly chosen high-power fields (×400) of the lung tissue. Immunoreactivity was categorized as negative (0, no positive cells), weak (+, <5%), moderate (++, 5–50%), or strong (+++, >50%). Quantitative analysis of cleaved caspase-3 and Bcl-2 expression was performed with ImageJ software (version 1.52f, NIH, Bethesda, MD, USA).

### 4.17. Statistical Evaluation

Data were analyzed using GraphPad Prism version 8. Multiple-group comparisons were performed using one-way analysis of variance (ANOVA) followed by Tukey’s post hoc test. Results are presented as mean ± standard deviation (SD), and differences were considered statistically significant at *p* < 0.05.

## 5. Conclusions

Alogliptin combined with amentoflavone more effectively reduced BLM-induced pulmonary fibrosis than either agent alone. This enhanced protection is probably due to the combined antioxidant, anti-pyroptotic, and anti-inflammatory actions of the two drugs, together with their coordinated modulation of the SIRT1/Nrf2/HO 1 axis and downregulation of TGF-β1 expression, leading to reduced oxidative damage, pyroptotic signaling, and fibrotic remodeling with restoration of autophagy/apoptosis balance in the lung tissue. Further investigations with larger animal cohorts are necessary to elucidate the molecular pathways underlying the effects of alogliptin and/or amentoflavone on BLM-elicited lung fibrosis. Future studies should employ advanced techniques such as Western blotting, mRNA expression analysis of key proteins, and knock-out animal models to strengthen the mechanistic insights. Moreover, evaluation of their role in BLM-induced lung injury within cancer-bearing models is crucial for understanding how these agents may influence signaling networks relevant to cancer biology.

## Data Availability

The original contributions presented in this study are included in the article. Further inquiries can be directed to the corresponding author.

## Abbreviations

The following abbreviations are used in this manuscript:

Bcl-2	Beta cell lymphoma-2
BLM	Bleomycin
HO-1	Heme oxygenase-1
GR	Glutathione reductase
GST	Glutathione S-transferase
IL-1β	Interleukin-1 beta
LC3-II	Microtubule-associated protein 1 light chain 3
MDA	Malondialdehyde
NLRP3	NOD-like receptor family pyrin domain-containing 3
Nrf2	Nuclear factor erythroid 2–related factor 2
P62	Sequestosome 1
SIRT1	Sirtuin-1
TGF-β1	Thioredoxin-interacting protein
